# Dose-dependent protective profile of *trans*-anethole in experimental traumatic brain injury in mice via modulation of apoptotic and inflammatory protein expression, and oxidative stress

**DOI:** 10.1590/acb408525

**Published:** 2025-11-10

**Authors:** Aziz Çevik, Burak Atlas, Gamze Erdoğan, Mehmet Salih Atama, Barış Altun, Tevfik Yılmaz

**Affiliations:** 1 Dicle University – Faculty of Medicine – Department of Neurosurgery – Diyarbakır – Turkey.; 2 Dicle University – Faculty of Medicine – Department of Histology and Embryology – Diyarbakır – Turkey.; 3 Genesis Private Hospital – Department of Neurosurgery – Diyarbakır – Turkey.; 4 Nusaybin State Hospital – Department of Neurosurgery – Nusaybin – Mardin – Turkey.

**Keywords:** Brain Injuries, Traumatic, Apoptosis, Inflammation, Oxidative Stress

## Abstract

**Purpose:**

To investigate the protective effect of *trans*-anethole in experimental traumatic brain injury (TBI) in mice.

**Methods:**

Thirty-five adult mice were divided into five groups (control, TBI, TBI+A40, TBI+A80, and TBI+A160). No treatment was performed in the control group. The treated groups (TBI+A40, TBI+A80, and TBI+A160) received 40, 80 or 160 mg/kg *trans*-anethole treatment, respectively. At the end of the experiment, the brains of the sacrificed animals were removed, and laboratory analyses were performed.

**Results:**

Malondialdehyde (MDA) levels in brain tissue of TBI and TBI+A40 were significantly increased, while MDA levels of TBI+A80 and TBI were similar. TBI+A160 and control tissue MDA levels were similar (p > 0.05), significantly different from TBI (*p* < 0.01). Immunodensity analyses showed that there was a significant difference between the control and TBI in terms of Bax, caspase 3, tumor necrosis factor-α (TNF-α) and interleukin- (IL)-1β immunoexpression. TBI+A40 immunoexpression was similar to TBI (*p* > 0.05), significantly different from the control groups (*p* < 0.05). In TBI+A80 and TBI+A160, pro-apoptotic Bax and caspase 3, pro-inflammatory TNF-α and IL-1β levels were also significantly improved. Immunoexpression levels of TBI+A160 and control were similar (*p* > 0.05).

**Conclusion:**

in our study, *trans*-anethole treatment had a dose-dependent neuroprotective potential against trauma-induced neurodegeneration.

## Introduction

Traumatic brain injury (TBI) is one of the major causes of morbidity and mortality that can be seen at any age^1^. The clinical features of TBI and the underlying complex pathophysiological features increase the value of preclinical studies and phase I/II studies. The success of these studies has led to the development of promising therapeutic approaches in relation to the severity of TBI. A therapeutic method other than surgical intervention for the treatment of TBI has not been developed yet^2^.

TBI-related injuries can be categorized as primary injury due to mechanical damage and secondary injury following this injury. Primary TBI may occur when different mechanical factors affect the brain at the same time. Primary TBI occurs because of neuronal damage provoked by a blow to the head and a series of neuroinflammatory events. Neuroinflammation is activated by increased microglial activity and depletion of mitochondrial glutathione, an important antioxidant compound. The decrease in glutathione and the accompanying intracellular calcium ion increase cause mitochondrial dysfunction leading to caspase activation and eventual apoptosis^3^^,^ 4.

Secondary damage following TBI is thought to result from multiple physiological processes, including free radical injury, inflammation, and glutamatergic excitotoxicity^5^,6. Studies have shown that the incidence of coexistence of both types of injuries is quite high in patients with moderate to severe brain injury; in addition, diffuse axonal damage has been shown in approximately 70% of cases with TBI^7^.

In the first 24 hours of the acute period following TBI, neutrophils, monocytes, and lymphocytes infiltrate into the damaged brain tissue due to the blood-brain barrier, being disrupted, and it has been demonstrated that pro-inflammatory interleukin (IL)-1β, IL-6 and tumor necrosis factor- (TNF)-α levels increase significantly in the acute period. The increase in TNF-α level, which is one of the members of Fas superfamily, causes interaction with Fas ligand and induction of apoptosis as a result of activation of caspases in brain tissue with TBI^8^. It has been reported that apoptosis occurs by activating caspase 3 in secondary TBI^9^.

Plant-derived antioxidants have the potential to be used in the treatment of many neurodegeneration-related diseases^10^^,^ 11. *Trans-*anethole is an essential oil found at high rates in fennel and aniseed plants, and there are scientific research results that it can be used in the treatment of different diseases^12^^–^ 14. The antioxidant, anticarcinogenic, anti-inflammatory and anti-hypernociceptive effects of *trans-*anethol have made this vegetable oil gain an important place in recent studies^15^.

For this reason, we investigated the protective effect of *trans-*anethol in TBI model in a dose-dependent manner in our current study, since it is a cheap herbal component and easy availability. In our literature review, there is no study that investigated the dose-dependent protective power of *trans-*anethol, which is an antioxidant component, in animals with TBI model. For this reason, we thought that this research has the potential to bring a new perspective to the TBI research topic.

## Methods

### Study design

This study was performed after receiving Experimental Animals Ethics Committee of Dicle University (approval number: 2022/40). The 35 adult BALB/c mice weighing between 28–30 g were equally divided into five groups (control, TBI, TBI+A40, TBI+A80, and TBI+A160). The animals in control group neither exposed to any application nor to a *trans-*anethol treatment.

TBI was induced in the TBI, TBI+A40, TBI+A80, and TBI+A160 groups by weight dropping model, as described by Zhang et al.^16^. In brief, the animals exposed to TBI were placed under general anesthesia through intramuscular (i.m.) administration of 80 mg/kg ketamine hydrochloride (Ketalar; Pfizer, Istanbul, Turkey) and 10 mg/kg xylazine hydrochloride (Rompun; Bayer Healthcare, Leverkusen, Germany), and TBI was induced by free weight dropping. Five-mg/kg dose of meloxicam was administered to all of the animals to control trauma associated pain and welfare of the mice. The doses of 40, 80, or 160 mg/kg *trans-*anethol was administered to the animals in the TBI+A40, TBI+A80, and TBI+A160 groups, respectively. The first dose of the *trans-*anethol was administered at the third hour, and the drug was administered by oral gavage at the same period for the following three days. At the end of the experiment, all animals in this study were sacrificed through exsanguination under general anesthesia of ketamine and xylazine. Total brain tissues were removed, and a portion of the damaged cerebral cortex was preserved in 10% formalin for light microscopic examination. The other part was frozen at -80°C for malondialdehyde (MDA) analysis.

### Measurement of tissue malondialdehyde level

Lipid peroxidation was measured from brain biopsy samples taken during the experimental study using the Esterbauer method. Twenty-one MDA reacts with thiobarbutyric acid at 90–95°C to form a pink colored chromogen. Fifteen minutes later, the samples were rapidly cooled, and the absorbance was read spectrophotometrically at 532 nm. The values obtained were expressed in nmol/g protein^17^.

### Tissue processing protocol

Brain tissues from experimental animals were subjected to a fixation procedure, and routine tissue processing protocol was applied^18^. They were then washed under water for 12 hours. For dehydration, they were kept in alcohols prepared in sequential ascending order for 1 hour. The tissues were then kept in a mixture of xylene and paraffin in equal proportions for 2 hours, and paraffin infiltration was performed. Then, the paraffin was replaced with clean paraffin, and this process was continued for another 1 hour. Tissue samples were placed on paraffin blocks. Microtome (catalogue no.: Leica RM2265, Wetzlar, Germany) was used to take 5-µm thick sections from these paraffin blocks. The sections were stained with hematoxylin and eosin (H&E) for histochemical analyses.

### Immunohistochemistry protocol

In sections taken from paraffin-embedded blocks, Bax (catalogue no.: sc-7480, Santa Cruz Biotechnology, Dallas, TX, United States of America), caspase 3 (catalogue no.: sc-56053, Santa Cruz Biotechnology, Dallas, TX, United States of America), TNF-α (catalogue no.: sc-52746, Santa Cruz Biotechnology, Dallas, TX, United States of America) and IL-1β (catalogue no.: sc-52012, Santa Cruz Biotechnology, Dallas, TX, United States of America) levels were examined through immunohistochemistry protocol^19^. For this purpose, the deparaffinized sections were passed through decreasing alcohol series, taken into distilled water and washed with buffered phosphate buffer (PBS). For antigen conversion, the samples were heated in citrate buffer (pH = 6), and then 3% H_2_O_2_ was added for endogenous peroxidase inhibition. Blocking solution (catalogue no.: TA-125-UB, Thermo Scientific, Waltham, MA, United States of America) was dripped onto the sections and incubated for 7 minutes at room temperature. Then, Bax, caspase-3, TNF-α and IL-1β antibodies were diluted at a ratio of 1:100 were added onto the sections and incubated overnight at 4°C. Tissue sections were then incubated with secondary antibody (catalogue no.: TP-125-BN, Thermo Scientific, Waltham, MA, United States of America) and peroxidase enzyme (catalogue no.: TS-125-HR, Thermo Scientific, Waltham, MA, United States of America). For the chromogenic reaction, ready-to-use 3,3’-diaminobenzidine (DAB) (catalogue no.: TA-125-HD, Thermo Scientific, Waltham, MA, United States of America) was used for colorimetric immunoreactivity. The samples counterstained with hematoxylin were then washed and passed through increasing series of alcohol and xylene and covered with entellan.

### Measurement of immunodensity

The DAB immunopositivity level of the immunohistochemically stained sections was counterstained with hematoxylin, and semiquantitative data were obtained. The staining intensity of the cerebral cortex sections of TBI animals and of the cerebral cortex region in the control group was evaluated. For this evaluation, the intensity of DAB positive areas in hematoxylin stain was calculated using Image J Version 1.53n software. These ratios were used for statistical analysis.

### Statistical analysis

Any difference between the groups was evaluated statistically. For this purpose, the results of Image J analysis were statistically evaluated by the Statistical Package for the Social Sciences (IBM) software. The data obtained were statistically subjected to the non-parametric Kruskal-Wallis test, and multiple comparisons between groups were made using the Tamhane T2 test. If the difference between the groups was *p* < 0.05, the difference between the groups was considered significant. The statistical results obtained were shown as mean ± standard deviation (SD).

## Results

### Tissue malondialdehyde results

According to the results of our study, MDA levels of TBI and TBI+A40 groups were similar. There was a significant difference between the MDA levels of the control group and TBI and TBI+A40 groups ^a-c^ *p* < 0.01. There was a significant difference between the MDA levels of the control group and TBI+A80 and TBI+160 groups ^a-b^ *p* < 0.01. It also showed that there was a significant difference between the MDA levels of TBI and TBI+A40 groups, TBI+A80 and TBI+A160 groups ^b-c^ *p* < 0.05. MDA analysis results showed that MDA levels, which increased after trauma, decreased significantly in the tissue as the *trans-*anethol dose increased. Detailed results showing the MDA levels between the groups are shown in Table 1.

**Table 1:** Tissue malondialdehyde (MDA) levels in control, traumatic brain injury (TBI), TBI+A40, TBI+A80, and TBI+160 groups*.

	MDA (nmol/g)
Control	8.62 ± 1.02^a^
TBI	17.04 ± 1.04^c^
TBI+A40	16.94 ± 0.75^c^
TBI+A80	14.10 ± 1.60^b.c^
TBI+A160	11.20 ± 2.27^a.b^

* The existence of different superscript on the MDA results demonstrates the significantly differences between the groups.

Similar symbol demonstrates the similarity:

a-b *p* < 0.01,

a-c *p* < 0.01,

b-c *p* < 0.05.

### Histopathologic results

**Figure 1:**
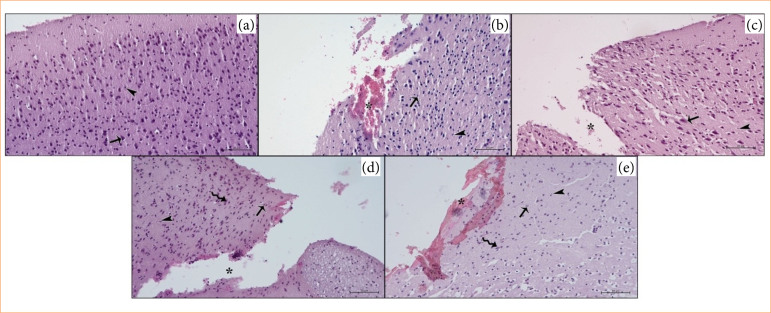
Histopathological representative micrographs in **(a)** control, **(b)** traumatic brain injury (TBI), **(c)** TBI+A40, **(d)** TBI+A80, and **(e)** TBI+A160 groups. Regular neuron and glial cells in control group. In TBI group, trauma associated tissue loss and hematoma, irregularity, and pyknosis in neurons and glial cells. In TBI+A40 group, the neural and glial cell degeneration severity was similar to the one in TBI group, but there was no bleeding on trauma portion of the cerebral cortex. In TBI+A80 group, the degeneration in neurons and glial cells improved around the traumatic portion of the cerebrum. The most success in neuroprotection is observed in TBI+A160 group. Neuron cells (arrow), neuroglial cells (arrowhead), traumatic brain region (asterisk), besides of the regular neuron, the degenerated neurons in d and e insets (curved arrow). Staining: hematoxylin and eosin. Bar: 20 µm.

Source: Elaborated by the authors.

In the brain samples of the control group, neuron cell bodies and glia cell bodies were found to have normal morphological structure. In the TBI group, intense hemorrhage in the injury areas of the frontal cortex, pyknotic nuclei in neuron cell bodies, and karyolysis, and an increase in perineural cavity were observed. Edema areas due to trauma were observed. Karyolysis and pyknotic nuclei were quite common in neuroglia cells. In the TBI+A40 group, hemorrhage, edematous tissue appearance, and tissue loss due to cerebral cortex damage were at the expected level. Pyknotic nuclei appearance in neuron cell bodies, cell morphology was generally irregular, and pyknotic nuclei appearance in neuroglia cells may be an important sign of apoptotic cell death. In the TBI+A80 group, areas of tissue loss, intense edema, and traumatic damage were observed in the traumatized cerebral cortex as expected. Nuclear destruction and pyknosis were observed intensely in neurons and glia cells. Especially in peritraumatic brain tissue pyramidal neurons, the pyramidal triangle image was preserved quite strongly. In the TBI+A160 group, in addition to hemorrhage in the trauma model regions of the cerebral cortex, blood tissue passed into the parenchyma, and intense edema was observed. Cortical neurons showed pyknotic nuclei and karyolitic neurons, glia cells showed intense cell degeneration and neurons with normal morphological structure. The representative micrographs of the cerebral sections of control, TBI, TBI+A40, TBI+A80, and TBI+A160 are shown in Fig. 1.

### Immunohistochemistry results

The results of the immunodensity statistical analyses of this study are shown in detail in Table 2. As a result of our immunohistochemical analyses, it was found that Bax immunodensity was quite low in the control group and significantly increased in the TBI group (*p* > 0.05), while in the TBI+A40 group, Bax immunodensity level was similar in TBI and TBI+A80 groups (*p* > 0.05) and significantly different from control and TBI+A160 groups (*p* < 0.05). Bax level in TBI+A80 group, which received moderate dose treatment, was similar to the one in TBI+A160 and control groups (*p* > 0.05). In TBI+A160 group, Bax immunodensity was similar to the one in control group (*p* > 0.05) and significantly different from TBI group (*p* < 0.05). Caspase-3 immunodensity was found to be low in the control group and significantly increased in the TBI group (*p* > 0.01). In the TBI+A40 group, caspase-3 immunodensity level was similar to the ones in TBI and TBI+A80 groups (*p* > 0.01) and significantly different from the control and TBI+A160 groups (*p* < 0.01). Caspase-3 level in TBI+A80 group was significantly different (*p* < 0.01) from TBI+A160 and control groups. In addition, caspase-3 immunodensity in TBI+A160 group was similar to the one in control group (*p* > 0.01).

**Table 2:** Statistical analysis results of tissue Bax, caspase 3, tumor necrosis factor (TNF)-α and interleukin (IL)-1β in control, traumatic brain injury (TBI), TBI+A40, TBI+A80, and TBI+A160 groups*.

	Bax immunodensity (%)	Caspase 3 immunodensity (%)	TNF-α immunodensity (%)	IL-1β immunodensity (%)
Control	15.58 ± 2.96^c^	10.94 ± 1.97^d^	9.99 ± 1.63^e^	11.27 ± 2.18^e^
TBI	24.75 ± 5.66^a^	15.75 ± 2.05^e^	22.46 ± 7.51^f^	17.83 ± 3.48^f^
TBI+A40	21.66 ± 5.67^a.b^	16.33 ± 2.74^e^	22.57 ± 8.55^f^	19.19 ± 5.04^f^
TBI+A80	18.99 ± 6.53^b.c^	14.87 ± 3.61^e^	16.16 ± 8.27^e.f^	17.47 ± 3.69^f^
TBI+A160	13.89 ± 2.21^c^	10.86 ± 1.69^d^	10.48 ± 1.77^e^	11.47 ± 2.36^e^

* Existence of different superscripts on each column indicate statistically significance between the groups.

The similar superscript demonstrates the similarity of the groups: ^a-b^ *p* < 0.05, ^a-c^ *p* < 0.05, ^d-e^ *p* < 0.01, ^e-f^ *p* < 0.001.

Source: Elaborated by the authors.

**Figure 2:**
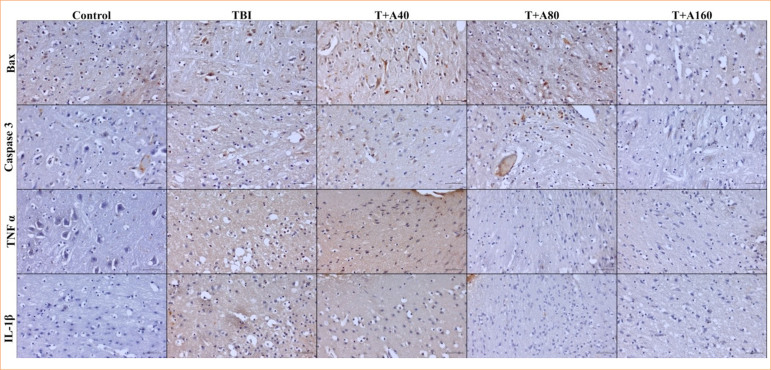
Representative immunoexpression micrographs of Bax, caspase-3, tumor necrosis factor (TNF)-α and interleukin (IL)-1β in control, TBI, TBI+A40, TBI+A80, and TBI+A160 groups. Staining: hematoxylin and eosin. Bar: 20μm.

Source: Elaborated by the authors.

**Figure 3:**
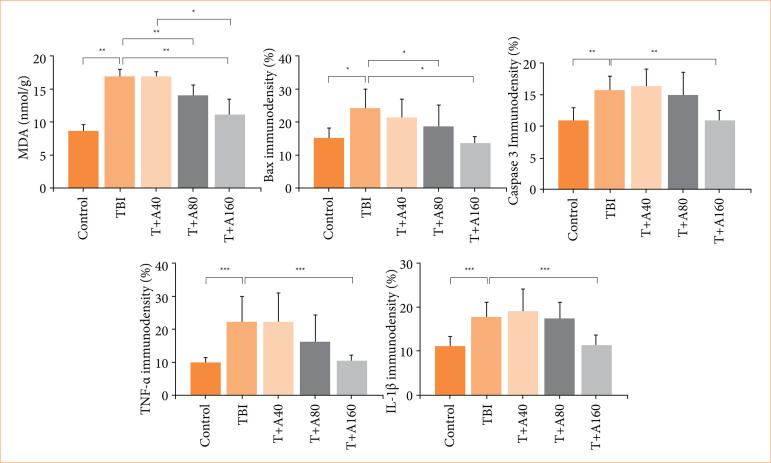
Graphical demonstration of the statistical analyses of tissue malondialdehyde (MDA) levels, Bax, caspase 3, tumor necrosis factor (TNF)-α and interleukin (IL)-1β immunodensity examination results. The symbols indicate statistically significance between the groups.

Immunohistochemistry micrographs of the groups are shown in Fig. 2. It was observed that TNF-α immunodensity was low in the control group, while there was intense expression in the TBI group (*p* > 0.05). In TBI+A40 group, TNF-α immunodensity level was similar to the ones in TBI and TBI+A80 groups (*p* > 0.05) and significantly different from control and TBI+A160 groups (*p* < 0.05). TNF-α level in TBI+A80 group was significantly different from TBI+A160 and control groups (*p* < 0.05), and TNF-α immunodensity in TBI+A160 group was similar to control group (*p* > 0.05). IL-1β immunoexpression level was low in the control group, whereas intense expression was observed in the TBI group (*p* > 0.05). IL-1β immunodensity level in TBI+A40 group was similar to TBI and TBI+A80 groups (*p* > 0.05) and significantly different from control and TBI+A160 groups (*p* < 0.05). IL-1β level in the TBI+A80 dose group was significantly different from TBI+A160 and control groups (*p* < 0.05), and IL-1β immunodensity in TBI+A160 group was similar to control group (*p* > 0.05). The graphical representation of the immunodensity statistical results of the groups is shown in Fig. 3.

*p < 0.05; **p < 0.01; ***p < 0.001. Source: Elaborated by the authors.

## Discussion

TBI is a clinical picture that occurs because of a complete or partial dysfunction of the central nervous system and is characterized by high mortality, morbidity, and disability^20^. In a study, it was reported that *trans-*anethol has a neuroprotective effect against rotenone-induced non-motor disorders and oxidative stress in Parkinson’s disease. Rotenone is said to cause neuroinflammation, hippocampal degeneration, and certain impairments in memory. Since rotenone-induced damage is thought to be caused by oxidative stress and inflammatory response, the use of antioxidant and anti-inflammatory agents is foreseen. It is known that rotenone-induced MDA levels increase in Parkinson’s patients and cause hippocampal neuron loss.

In this study, it was shown that high-dose anethol 250-mg/kg treatment improved cognitive functions and significantly decreased hippocampal MDA levels. In addition, it was observed to increase the survival number of neurons in the hippocampal region after high-dose treatment. In the light of these findings, anethol was shown to have both neuroprotective and antioxidant effects against non-motor damage caused by rotenone^21^.

As a result of our studies, it was shown that the MDA levels of TBI and TBI+A40 groups were similar, but there was a difference between the MDA levels of TBI, TBI+A40, TBI+A80, TBI+A160, and the control group. There was a significant decrease in the MDA levels, which increased after trauma in the anethol-treated groups.

In the histopathological study^22^, no nerve tissue damage was observed in the brain cortex of all rats in the control group. Neurons and neuroglia cells were said to have normal morphological characteristics. In the first-, sixth- and 24^th^-hour trauma groups, cortical neurons showed cytoplasmic eosinophilia called eosinophilic neurons and pyknotic nuclei without discernible nucleolus. Perineural satellitosis, in which multiple glia cells accumulate around the eosinophilic neurons, was also observed. The fibrillar matrix of the cerebral cortex (neurofil) showed edema-induced spongiosis of the cellular extensions of neurons and glia cells. In our study, neuron cell bodies and glia cell bodies forming the structure of brain samples taken from the control group were found to have normal morphological structure. In the TBI group, intense hemorrhage in the injury areas of the frontal cortex, pyknotic nuclei in neuron cell bodies, and karyolysis and an increase in perineural cavity were observed in brain samples. In TBI+A40 group, hemorrhage and edematous tissue image were observed due to cerebral cortex damage. Pyknotic nuclei were observed in neuron cell bodies. In the TBI+A80 group, areas of tissue loss and intense oedema were observed in the traumatized cerebral cortex, and in the TBI+A160 group, blood tissue passed into the parenchyma, and intense edema was observed in the trauma modeled regions of the cerebral cortex. Pyknotic nuclei, karyolytic neurons, and intense cellular degeneration in glia cells were observed in cortical neurons.

In another study, findings on the strong antioxidant potential of anethol and data suggest that it may be protective in cardiac ischemia reperfusion injury. In this study, although the cardiac TNF-α level of animals exposed to ischemia showed a significant increase, it was found that the cardiac content of TNF-α level significantly decreased in the groups given 100- and 200-mg/kg doses^23^. In our study, we revealed that there was a significant increase in TNF-α level in the TBI group, but a significant decrease in TNF-α level occurred especially in the groups given TBI+A80 and TBI+A160 doses.

In a study, it was determined that anethol decreased intestinal barrier dysfunction and intestinal inflammation caused by necro-hemorrhagic enteritis in broilers. At the same time, they revealed that ileal Bax and jejunal caspase-3 increased the apoptotic index and mRNA expressions of ileal Bax and jejunal caspase-3 and decreased the mRNA levels of jejunal Bcl-2 in the necro-hemorrhagic enteritis group, but these effects were eliminated with 600-mg/kg anethol administration. In addition, they reported that there was no significant difference between the treatment groups in terms of ileal apoptotic index, mRNA levels of Bcl-2 and caspase-3, and jejunal Bax^24^.

In our study, pro-apoptotic Bax level was also analyzed. We think that the significant increase in Bax immunoexpression level in the TBI group compared to the control group is due to the reflex protection mechanism of the organ against TBI. In addition, the significant decrease in Bax expression level compared to the anethol-treated groups supports this hypothesis and that anethol, which is known to have antioxidant potential, may protect the organ against TBI.

In this study, it was also revealed that the serum concentrations of TNF-α and IFN-γ were significantly increased in the group with necro-hemorrhagic enteritis, and especially the production of these cytokines was significantly decreased by 600-mg/kg anethol administration. They also revealed that ileal IL-4 and jejunal IL-10 showed lower mRNA expressions compared to the anethol treatment group with necro-hemorrhagic enteritis. In the light of all these findings, they suggested that 600-mg/kg anethol administration may treat necro-hemorrhagic enteritis by modulating the production of pro-inflammatory and anti-inflammatory cytokines^24^. In our study, it was similarly demonstrated that the amount of TNF-α, Bax, caspase-3, and IL-1β decreased significantly compared to the trauma group, but mRNA expressions and IFN-γ levels were not examined in our study.

In another study, the effects of 125- and 250-mg/kg doses of anethol before middle cerebral artery occlusion were investigated. It was observed that some neurons in the rat cortex and hippocampal region in the occlusion group were heterogeneously distributed, the cell body was shrunken and extremely irregular, the cytoplasm was red-stained, the nucleus was pyknotic, and the structure was unclear. However, the groups treated with anethol before the procedure showed a significant decrease in the number and degree of neurons in the rat cortex and hippocampus compared to the occlusion group, as well as a significant improvement in degeneration, necrosis, and loss. In addition, there was a significant increase in MDA, superoxide dismutase (SOD), and catalase content in the occlusion group, whereas in the groups given anethol before the procedure, there was a significant decrease in MDA content, as well as SOD and catalase amounts.

In the other parameters analyzed, it was reported that inflammatory cytokines (IL-1ß, IL-6, and TNF-α) were significantly increased in the occlusion group, and the increases of these inflammatory cytokines were reversed in the anethol-treated groups. As for apoptotic markers, it was revealed that a decrease in the gene expression of Bcl-2 occurred in association with an increase in the gene expression of Bax in the occlusion group, while these changes were reduced in the anethol-treated group^s^ 25. In our study, SOD and catalase levels could not be analyzed, but the decrease in cytokine levels and Bax levels were consistent with our experiments.

In the literature, both experimental animal studies and clinical studies investigating the antioxidant and anti-inflammatory effects of anethole are very limited. There are limited number of experimental studies investigating the neuroprotective efficacy of anethole. Therefore, the importance of our study increases, and more detailed new studies are needed. Especially in this experimental study, we emphasized this neuroprotective property of anethole and tried to draw attention to this aspect, because we think that anethole has strong neuroprotective effects due to its antioxidant and anti-inflammatory properties, and more studies are needed in this direction.

### Limitations of this study

Results of this study indicated that *trans-*anethole might bear a very promising neuroprotective agent in TBI in mice. Although our morphological and immunohistochemical examinations showed that the increased inflammatory and apoptotic protein expression levels associated with TBI can be improved through *trans-*anethol treatment, the result of this study expressed acute observations and promising property of *trans-*anethol in TBI in mice.

The lack of laboratory analysis methods such as polymerase chain reaction and Western blot limited the observations of this study. In addition, nerve tissue and/or glia cell specific markers such as BDNF, GFAP, and s100β could be used to validate the reliability and rationality of these results and even would be addressed to understand the underlying potential neuroprotective profile of *trans-*anethol in TBI. Lack of the mentioned methodologies and gene/protein analyses is a limitation of this study.

Although there are some limitations, as mentioned above, this is one of the first studies reporting neuroprotective potential of *trans-*anethol in TBI through regulating inflammatory and apoptosis associated protein expressions which indirectly impact on the cellular morphology in central nervous tissue. For that reason, we believe more, and detailed new studies are required to understand the underlying mechanisms and measure the strength of *trans-*anethol in TBI to understand clinical applicability of this safe plant sourced drug.

## Conclusion

Results of this study indicated that *trans-*anethole treatment during post-traumatic period has a potential to improve tissue MDA level, and generation of inflammatory cytokines of TNF-α, IL-1β, and apoptotic proteins such as Bax and Caspase 3 because of TBI. These mentioned regulatory profile of *trans-*anethole has mainly observed in higher dose treated animals, while there was not any significantly change in the lowest dose treated animals. For that reason, neuroprotective potential of *trans-*anethole might be related to various and different factors.

When the literature is searched, we have not reached any study that examined the potential of *trans-*anethole in TBI. Although this study is expressing promising results, detailed and more studies are required to understand the underlying mechanisms and explore the effective dose range of this drug.

## Data Availability

The generated data during examination of this study were available from the corresponding author upon a reasonable request.
